# The Maastricht Ultrasound Shoulder pain trial (MUST): Ultrasound imaging as a diagnostic triage tool to improve management of patients with non-chronic shoulder pain in primary care

**DOI:** 10.1186/1471-2474-12-154

**Published:** 2011-07-08

**Authors:** Ramon PG Ottenheijm, Manuela A Joore, Geert HIM Walenkamp, René E Weijers, Bjorn Winkens, Jochen WL Cals, Rob A de Bie, Geert-Jan Dinant

**Affiliations:** 1 Dept. of General Practice, CAPHRI School for Public Health and Primary Care, Maastricht University, PO Box 616, 6200 MD Maastricht, The Netherlands; 2 Dept. of Clinical Epidemiology and Medical Technology Assessment, Maastricht University Medical Centre, Maastricht, The Netherlands; 3 Dept. of Orthopaedic Surgery, Maastricht University Hospital, Maastricht, the Netherlands; 4 Dept. of Radiology, Maastricht University Medical Centre, Maastricht, The Netherlands; 5 Dept. of Methodology and Statistics, CAPHRI School for Public Health and Primary Care, Maastricht University, Maastricht, the Netherlands; 6 Dept. of Epidemiology, CAPHRI School for Public Health and Primary Care, Maastricht University, Maastricht, The Netherlands

## Abstract

**Background:**

Subacromial disorders are considered to be one of the most common pathologies affecting the shoulder. Optimal therapy for shoulder pain (SP) in primary care is yet unknown, since clinical history and physical examination do not provide decisive evidence as to the patho-anatomical origin of the symptoms. Optimal decision strategies can be furthered by applying ultrasound imaging (US), an accurate method in diagnosing SP, demonstrating a clear relationship between diagnosis and available therapies. Yet, the clinical cost-effectiveness of applying US in the management of SP in primary care has not been studied. The aim of this paper is to describe the design and methods of a trial assessing the cost-effectiveness of ultrasound imaging as a diagnostic triage tool to improve management of primary care patients with non-chronic shoulder pain.

**Methods/Design:**

This randomised controlled trial (RCT) will involve 226 adult patients with suspected subacromial disorders recruited by general practitioners. During a Qualification period of two weeks, patients receive care as usual as advised by the Dutch College of General Practitioners, and patients are referred for US. Patients with insufficient improvement qualify for the RCT. These patients are then randomly assigned to the intervention or the control group. The therapies used in both groups are the same (corticosteroid injections, referral to a physiotherapist or orthopedic surgeon) except that therapies used in the intervention group will be tailored based on the US results. Ultrasound diagnosed disorders include tendinopathy, calcific tendinitis, partial and full thickness tears, and subacromial bursitis. The primary outcome is patient-perceived recovery at 52 weeks, using the Global Perceived Effect questionnaire. Secondary outcomes are disease specific and generic quality of life, cost-effectiveness, and the adherence to the initial applied treatment. Outcome measures will be assessed at baseline, 13, 26, 39 and 52 weeks after inclusion. An economic evaluation will be performed from both a health care and societal perspective with a time horizon of 52 weeks.

**Discussion:**

The results of this trial will give unique evidence regarding the cost-effectiveness of US as a diagnostic triage tool in the management of SP in primary care.

## Background

With up to 100 new patient encounters per general practitioner (GP) per year, and a prevalence of 17-20% in the general population, shoulder pain (SP) is a common and sizable problem in primary care [1-4]. About 70% of the patients with a new episode of SP show incomplete recovery within six weeks, 50% report persistent complaints after six months, and 40% are not recovered after one year [4]. Troublesome pain is the most prominent symptom in adult patients with SP until the age of 65 years [5]. Prolonged and recurrent pain episodes result in frequent consultations[6]. Within one year, 40% of the patients with SP have at least one reconsultation with the GP [4]. Roughly 30% of the patients with SP report limitations in daily life and sick leave is common [3]. It is suggested that prolonged and recurrent episodes generate substantial costs for care and sick leave [7]. In general, indirect costs, such as costs caused by sick leave from paid work, are substantial and represent a higher burden to the economy than direct costs [8]. Hence, there is a great need to improve diagnosis and prognosis from both the individual patient perspective as well as from a societal perspective.

Subacromial disorders are considered to be the most common pathology affecting the shoulder. In 80% of the cases with SP in primary care, the rotator cuff is the prominently affected anatomical structure [4]. The spectrum of subacromial pathology is extensive and includes rotator cuff tendinopathy (tendinosis), calcific tendinitis, partial- or full-thickness tears, and acute or chronic subacromial-subdeltoid bursitis [9-11]. In primary care, the prevalence of these disorders are unknown. Studies in secondary care have shown a prevalence ranging from 30-39% for tendinopathy, 13-15% for calcific tendinitis, 13-51% for partial-thickness tears, 24-70% for full-thickness tears, and 12-56% for bursitis [12]. Conflicting theories have been proposed to explain the mechanisms leading to pathology, and more research is needed to understand the disease process [10,13,14]. However, the dominant view is that many shoulder complaints have their origin in a dynamic pathology, with subacromial impingement as the initial stage, and rotator cuff tears as the final stage [15,16].

For all these specific subacromial disorders, specific therapies are available [9,17]. After the mainstay of paracetamol or non-steroidal anti-inflammatory drugs (NSAIDs), tendinopathy preferably is treated with physiotherapy, calcific tendinitis and bursitis with subacromial corticosteroid injections, partial-thickness tears with physiotherapy, and in case of full-thickness tears surgery should be considered. Unfortunately, physical examinations used to evaluate the various disorders are fraught with uncertainty [4,10,18]. As a result, the diagnostic phase does not often lead to a patho-anatomical diagnosis. Therefore, the guideline for SP of the Dutch College of General Practitioners (DCGP) advises GPs to start treatment based on patients' signs and symptoms rather than on a suspected patho-anatomical diagnosis. The advised treatment for all patients consists of a stepwise approach, which starts with advice and paracetamol or NSAIDs for 2 weeks. In persisting cases subacromial corticosteroid injections and referral to a physiotherapist are advised, depending on the level of pain and functional limitations respectively. Referral to a specialist or imaging modalities are advised if these usual care treatments fail [4].

The current diagnostic strategy leads to a substantial case mix, and the stepwise treatment approach is expected to dilute the effects of the indicated interventions in the total population considerably. This approach delays specific therapy tailored to the pathology, which is remarkable knowing that a more effective approach to SP is available, which can lead to a better prognosis and less costs. We hypothesise that the current stepwise approach can be improved by giving the GP more evidence as to the patho-anatomical origin of the symptoms of SP. Due to the sufficient diagnostic accuracy [12,19,20] and clear relationship between US diagnosis and available evidence based therapies, applying US in the management of SP in primary care can solve this problem. US has the advantage of being non-invasive, relatively inexpensive, and producing high-resolution dynamic images of the shoulder. However, additional costs of management with US in patients with SP should be balanced by an increase in patients' health status and/or monetary savings for society. This cost-effectiveness, which has not been studied before, is the subject of our trial. This study foresees in the evidence gap that is addressed in the SP guideline of the DCGP.

Therefore, the primary objective of the present study is to investigate the effects of diagnostic US and its related tailored treatment decisions on clinical recovery and costs (cost-effectiveness) compared to usual care in individuals with non-chronic SP (pain less than 3 months) in primary care. The secondary objectives of the study are: (i) to investigate the effects of diagnostic US and its related treatment decisions on shoulder pain, performance of daily activities, and health-related quality of life compared to the usual care; (ii) to determine the prevalence of the specific subacromial disorders based on US reports; (iii) to evaluate to what extent the introduction of US influences management decisions, what type of treatment is provided to what type of patient (patient characteristics), and the adherence to the initially applied treatment.

## Methods/design

### Design

**Figure 1:**
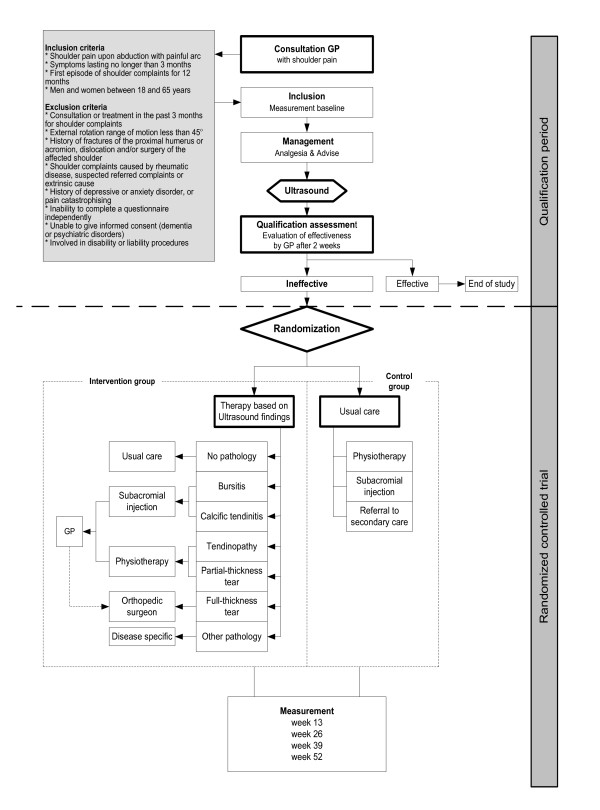
Flowchart of the study

In a pragmatic design, which initially follows the guideline for SP of the DCGP[4], a study consisting of two phases will be carried out: a Qualification Period of two weeks followed by a randomised controlled trial (RCT) with a 50 week follow-up period (Figure 1). The Qualification period aims to filter out patients with a favorable natural course. During the 2-week Qualification period all patients are advised to start with paracetamol or NSAIDs in maximum dosage on a time contingent base, receive advice regarding activities of daily living, work, hobbies and sports. This advise fits within the first line treatment as recommended in the guideline for SP of the DCGP. Moreover, patients are referred for US of the shoulder to the radiology department of the Maastricht University Medical Centre (MUMC) or Orbis Medical Centre (OMC) in Sittard-Geleen, The Netherlands. Based on the qualification assessment at 2 weeks, patients with insufficient improvement qualify for the RCT. These patients are randomly assigned to the intervention or the control group. The therapies used in both groups are the same except that therapies used in the intervention group will be tailored based on the US results. Primary and secondary outcome measures will be assessed at baseline, 13, 26, 39 and 52 weeks after inclusion. Patient recruitment started in November 2010 and patients will be included until October 2012. The Medical Ethics Committee of the Maastricht University Medical Centre has approved this protocol (NTR2403). This trial is officially called the Maastricht Ultrasound Shoulder pain Trial (MUST).

### Setting

Patients for this trial will be recruited and treated by 21 GPs, working in 11 general practices, in the Westelijke Mijnstreek, a region in the southern part of the Netherlands. A total of 80 general practitioners received a letter inviting them to participate in this study. Of them, 21 GPs agreed to participate in the study. They attended a 2-hour instruction workshop with their practice assistants. This workshop provided information about the guideline for SP of the DCGP, the inclusion and randomisation procedures, as well as the interventions to be applied. All participating GPs were asked to give the names of their preferred physiotherapy practices. These physiotherapists were additionally invited for the workshop. In total 26 physiotherapists from 12 of the 14 invited physiotherapy practices attended the workshop. The physiotherapists were presented with an evidence based statement regarding subacromial disorders in a separate 1-hour parallel program [21]. Those two practices not represented, received a handout of the presentation and study materials.

### Study population

The study population will comprise of patients with SP, who are physically active with troublesome pain, and visit their GP with an episode of SP. To be eligibly for recruitment patients have to fulfil the following eligibility criteria: (i) shoulder pain upon abduction with painful arc; (ii) symptoms lasting no longer than three months; (iii) first episode of SP for 12 months; (iv) age between 18 and 65 years. Exclusion criteria will be: (i) consultation or treatment for SP in the past three months; (ii) glenohumeral external rotation range of motion less than 45 degrees as this is a reason to suspect a glenohumeral disorder like osteoarthritis or a frozen shoulder; (iii) history of fractures of the proximal humerus or acromion, dislocation and/or surgery of the affected shoulder; (iv) shoulder complaints caused by rheumatic disease, suspected referred complaints or extrinsic cause; (v) history of depressive or anxiety disorders, or pain catastrophising; (vi) inability to complete a questionnaire independently; (vii) unable to give informed consent (dementia or psychiatric disorders); (viii) involved in disability or liability procedures.

### Interventions

Before randomisation, US of the shoulder is performed by a radiologist with 8 to 20 years of experience in musculoskeletal US at the MUMC or OMC using a protocol-based scanning approach (Additional file 1) [22-26]. US is an accurate diagnostic instrument to diagnose subacromial disorders [12]. The distinguishable disorders are tendinopathy, calcific tendinitis, partial and full thickness tears, and subacromial bursitis.

#### Intervention group

The advised evidence based, tailored treatment steps are described in Additional file 2 [4,9,17,27-36]. To prevent treatment of supposed asymptomatic pathology, GPs will link US pathology to history and findings from physical examination. In case pathology other than rotator cuff disorders is diagnosed, it will be treated according to this diagnosis (e.g. in cases of signs of rheumatoid arthritis patients are referred to a rheumatologist). If there is no detectable pathology, usual care according to the guideline for SP of the DCGP will be advised [4]. In cases where multiple US findings are present, the most relevant abnormality will be selected by the GP on the basis of the clinical findings. With an explanation, and within the recommendations made in the guideline for SP of the DCGP, GPs are allowed to deviate from the advised treatments steps. The treatments are standardised.

#### Control group

Usual care according to the guideline for SP of the DCGP will be applied in the control group. It consists of a pragmatic, stepwise approach; a wait-and-see policy with advice and analgesia for another 2 weeks; in persisting cases corticosteroid injections and referral to a physiotherapist are advised, depending on the level of pain and functional limitations respectively; referral to a hospital specialist is advised if conservative treatment fails [4].

### Randomisation and allocation

Based on the qualification assessment at 2 weeks, unrecovered patients (measured by the Global Perceived Effect questionnaire; see below) qualify for the RCT and are randomly assigned by central block randomisation (blocks of 4) to the intervention or control group after stratification for age (≥ 50 years). Neither the patient nor the GP can be blinded for the allocated treatment. However, US results are only disclosed to GPs of those patients in the intervention group, as well as those patients themselves. In case a patient is allocated to the intervention group, the GP receives the US result and the advised corresponding treatment strategy. In the control group, neither the patients nor the GPs receive the US results. Their US results will be presented to the GP at the end of patients' follow-up period.

The radiologist performing the US, is not allowed to communicate with the patients about the US findings and results. In case a fracture, septic bursitis or arthritis, or a life-threatening disorder (e.g. tumour) is diagnosed, the radiologist will immediately inform the GP with c.c. to the investigator, and the patient will be excluded and not randomised.

### Outcome assessment

At baseline, demographic information will be collected including age, sex and profession, as well as disease specific information regarding the affected side, onset, duration of symptoms, possible cause of complaints, history of shoulder complaints, neck complaints and dominant arm. A number of outcome measures will be collected at baseline, 13, 26, 39 and 52 weeks after inclusion (Table 1).

**Table 1:** Outcome measures

Primary outcome	**T**_**0**_	**T**_**1**_	**T**_**2**_	**T**_**3**_	**T**_**4**_
Patient-perceived recovery (GPE)		**+**	**+**	**+**	**+**

**Secondary outcomes**					

Performance of daily activities (SDQ)	**+**	**+**	**+**		**+**
Shoulder pain (SPS)	**+**	**+**	**+**		**+**
Quality of daily life (EQ-5D)	**+**	**+**	**+**		**+**

**Other measures**					

Costs		**+**	**+**	**+**	**+**
Number of re-consultations, corticosteroid injections, diagnostic imaging procedures, referrals to physiotherapy and hospital					**+**

Abbreviations used: T_0 _- baseline; T_1 _- 13 weeks; T_2 _- 26 weeks; T_3 _- 39 weeks; T_4 _- 52 weeks; GPE - Global Perceived Effect; SDQ - Shoulder Disability Questionnaire; SPS - Shoulder Pain Score; EQ-5D - Euroqol five-item quality of life questionnaire

#### Primary outcome measure

The primary outcome measure for the clinical effectiveness is the patient-perceived recovery using the Global Perceived Effect questionnaire (GPE)[37]; a one-item score concerning recovery following treatment, measured on a seven-point ordinal scale. Patients are considered to be recovered when they report to be much improved or fully recovered. Together with disease-specific functional status measures, this is considered to be an important outcome variable for shoulder complaints.

#### Secondary outcome measures

##### Shoulder Pain Score (SPS)

The SPS is a questionnaire to assess pain experienced by patients with shoulder disorders and includes a 24-hour recall frame. The score consists of six pain symptom questions and a 10-point Scale [38]. The SPS has been proved to be a useful instrument for following the course of the disorder over time, and gives an indication when a patient feels cured. Each question receives a maximum of four points. The VAS is also transposed to a four-point scale (0 = 1, 1-3 = 2, 4-6 = 3, 7-10 = 4). The minimum SPS score is seven points, the maximum score 28.

#### The Shoulder Disability Questionnaire (SDQ)

The SDQ assesses the performance of daily activities. This variable will be assessed by a 16-item questionnaire for functional status limitation in patients with shoulder disorders and assesses the past 24 hours [39]. The 16 questions can be answered with either yes, no or not applicable. The final SDQ-score will be calculated by dividing the number of positive responses by the total number of applicable items, and multiplying this score by 100. Consequently, the SDQ-score can range from 0 to 100 with a higher score indicating more severe disability.

#### The Euroqol five-item quality of life questionnaire (EQ-5D)

The EQ-5D is one of the most commonly used generic (that is not disease specific) measures used to quantify the health related quality of life in people with musculoskeletal disorders [40,41]. It is a patient-reported measure that consists of two sections. The first section comprises five questions with three levels of severity in each (1 = no problem, 2 = moderate problem, 3 = severe problem) that covers five dimensions of health: mobility, self-care, usual activities, complaints/discomfort, and anxiety/depression. This generates 243 theoretically possible health states. Calculation of the index score will be performed according to the European recommendations [42]. The second section is a visual analogue scale ranging from 0 (worst imaginable health state) to 100 (best imaginable health state).

#### Costs

Intervention costs, direct and non-health care costs, as well as indirect costs will be collected. A questionnaire composed of 24 questions regarding resource use and expenses in the last three months will be used. In addition, the research team will contact the GPs, physiotherapists, and hospital specialists in case patients have been referred, for treatment costs [43]. Standard unit cost data will be derived from reliable published sources [44]. The costs related to the intervention itself (application of US) will be assessed by detailed cost pricing.

#### Other assessments

Also in addition, it will be evaluated to what extent patients are blinded for the US findings and results prior to randomization, to what extent the introduction of US influences management decisions, what type of treatment is provided to what type of patient (patient characteristics), and what adherence rates to the initial applied treatment can be shown.

### Sample size

The sample size calculation is based on a recovery rate (measured by GPE) of 60% in the control group [4] and 80% in the intervention group after 52 weeks, a two sided-alpha of 0.05, a statistical power of 0.80, and a drop-out rate at 52 weeks of 10% [45,46]. We need 90 patients per study group to detect the difference of 20% between the study groups after 52 weeks; the minimal clinical important difference. With the expectation that the Qualification period filters out 20% of the patients, we need to include 226 patients. With up to 100 new patient encounters per GP per year [1-4] and approximately 25 eligible patients per GP per year, two years of recruitment, 50% consent to the study, and 50% drop-out for other reasons, we need 20 GPs to participate to include the required 226 patients. Based on the reported prevalence, and our experience, we expect to encounter enough patients with symptomatic US pathology to complete recruitment within two years.

### Data analysis

The primary analysis will be intention-to-treat and will compare the patient-perceived recovery measured by the Global Perceived Effect questionnaire (GPE) at 52 weeks after randomisation of patients managed by US tailored treatment (intervention group) and those having received care as usual (control group). In order to study the influence of protocol violations on the study outcomes, a per protocol analysis will be performed. Patients with documented deviations from the study protocol (that is no adherence to the treatment steps mentioned in Additional file 2) will be excluded from this analysis.

Continuous variables will be presented as mean ± standard deviation and categorical variables as number (%). The longitudinal trend of primary and secondary parameters will be compared between the intervention and control group using logistic and linear mixed models to take into account the dependency of repeated measurements and nesting structure of data (patients within GP practices). Baseline characteristics that a priori are considered to be possible prognostic factors (fast or gradual development of SP, possible cause of SP, dominant shoulder affected, concomitant neck complaints, and physical work with upper extremities), will be included in the mixed models.

An economic evaluation will be performed from both a health care and societal perspective with a time horizon of 52 weeks. The incremental cost-effectiveness ratios will be expressed as the costs per additionally recovered patient (from a health care perspective) and the costs per Quality Adjusted Life Years (from a societal perspective). Sensitivity analyses will be performed to assess the influence of relevant factors. Finally, bootstrap analysis will be performed to quantify the uncertainty surrounding the incremental costs and effects. Based on these results, a Cost-Effectiveness Acceptability Curve will be constructed to show the probability that the intervention is cost-effective.

## Discussion

This study uses a randomised controlled trial design to investigate whether US as a diagnostic triage tool is more effective on clinical recovery and is more cost-effective than continuation of usual care in patients with non-chronic SP in general practice. This study foresees in the evidence gap that is addressed in the SP guideline of the DCGP.

Primary inclusion criterion is the GP's suspicion of a subacromial disorder being the primary cause of SP. GPs are daily facing patients with SP and, based on the guideline for SP of the DCGP, they are able to differentiate between subacromial, glenohumeral and extrinsic disorders for SP. The pragmatic approach of this trial leaves them very close to daily practice when considering recruiting patients for this study.

This study is designed as a randomised controlled trial. However, since blinding patients is not possible in this pragmatic study, information bias has to be taken into account. As a stratagem to prevent this, radiologists are not allowed to discuss the US findings and results with patients. To evaluate this information bias, we included a question in the questionnaire at 13 weeks. Patients allocated to the control group and their GPs do not receive the US result until the end of patients' follow-up period.

The treatment options for the intervention and control group do not differ significantly. However, the significant difference between the treatment regimes in the intervention and control groups is that patients in the intervention group receive tailored treatment according to their patho-anatomical disease state. This in contrast with patients in the control group, who are treated according to the guideline SP of the DCGP, and receive treatment based on complaints. In this cost-effectiveness study we compare diagnosis - treatment combinations, and not only treatments. It is our hypothesis that these tailored treatment regimes, applying evidence based treatment without delay, have a positive effect on patient recovery and costs. Moreover, the prevention of unnecessary interventions can contribute to this effect.

In general, the accuracy of US is operator dependent. In experience hands US is an accurate diagnostic instrument to diagnose subacromial disorders [12]. For study purposes one can chose for a single operator or multiple operators per radiology department. For two reasons we choose for multiple operators. First, due to our design (the US has to be made within two weeks after patient inclusion, and the inclusion period runs for two years), the radiology departments were not able to guarantee a single operator. Second, a single operator would limit the external validity, as in The Netherlands it is common that within a radiology department, more radiologists are experienced in musculoskeletal ultrasound. Therefore, all US in our study are only made by experienced radiologists in musculoskeletal ultrasound. For study purposes, these radiologists use a standardized protocol (Additional file 1), which was developed by the project group and radiology departments.

It is likely that degeneration is one of the mechanisms leading to rotator cuff pathology [14]. As degeneration is part of the normal aging process, and therefore a potential confounder, we choose to stratify for age. In this respect, we realise that degeneration is a continuum, and evidence for a clear cut-off point for age as a confounder is lacking. Although arbitrary, we think it is plausible to set this cutt-off point for age at 50 years.

Rotator cuff tears are also common in asymptomatic and unselected populations [47,48]. The incidence of tears is known to increase with age and can be considered as part of the normal ageing process [47]. In this respect, we realise that overdiagnosis is to be avoided. However, the current diagnostic strategy leads to underdiagnosis. We do realise that in general a certain percentage of US recorded pathology is not directly responsible for the reported symptoms. Therefore, to prevent treatment of asymptomatic pathology, US pathology will be linked to medical history and physical examination.

As the prevelances of the different subacromial disorders in primary care are unknown, it is questionable whether we will encounter enough patients to start tailored treatment in the intervention group. Based on the reported prevalences in secondary care, and our experiences with patients in primary care who underwent shoulder US, we expect to encounter enough patients with different symptomatic US pathologies to start tailored treatment.

Physiotherapists generally utilise a variety of techniques such as mobilization, soft tissue massage, exercises and education. Often, their program is based on their own experiences. To ensure a consistent program, we trained the physiotherapists in an evidence based approach for subacromial disorders (released by the Royal Dutch Society for Physical Therapy) [21].

This study will ensure that the cost-effectiveness of US as a diagnostic triage tool in primary care is adequately evaluated. It will fill the gaps in the current evidence base and may guide clinical practice and policy. Importantly, the results obtained can be used to formulate (new) guidelines. In the Netherlands, guidelines of the DCGP, including the one for SP, are seen as the "gold standard" for the management of frequently occurring diseases and health problems. If US is considered cost-effective, this pragmatic study will contribute to dissemination. This potentially reduces unnecessary interventions, reconsultations, and referrals to physiotherapists and hospital. Referrals will be based on more adequate questions. If this is a negative trial, then resources will be saved by not promoting US in the early management of SP. Overall, quality of care in patients with SP will improve.

## List of abbreviations

DCGP: Dutch College of General Practitioners; EQ-5D: Euroqol five-item quality of life questionnaire; GP: General practitioner;GPE: Global Perceived Effect; MUMC: Maastricht University Medical Centre; MUST: Maastricht Ultrasound Shoulder pain Trial; NSAIDs: Non-steroidal anti-inflammatory drug; OMC: Orbis Medical Centre in Sittard-Geleen; RCT: Randomised controlled trial; SDQ: Shoulder Disability Questionnaire; SP: Shoulder pain; SPS: Shoulder pain score; US: Ultrasound imaging

## Competing interests

RO is member of the Supervisory board of MCC Omnes, Sittard, The Netherlands (Diagnostic and transmural care centre in the Westelijke Mijnstreek region). The other authors declare that they have no competing interests.

## Authors' contributions

RO, GD and RB originated the idea for the study. RO, GD, RB and GW designed the trial protocol. RO and RW designed the US protocol. RO and MJ designed the economic analysis. RO and BW designed the statistical analysis. RO drafted the manuscript and the other authors have read and corrected draft versions of the manuscript and approved the final manuscript.

## Pre-publication history

The pre-publication history for this paper can be accessed here:

http://www.biomedcentral.com/1471-2474/12/154/prepub

## Supplementary Material

Additional file 1 **Ultrasound examination technique and diagnosis**. Specification of the US technique and the criteria for diagnosis Click here for file

Additional file 2 **Ultrasound diagnosis tailored treatment steps**. specification of the evidence based tailored treatment steps Click here for file
