# Supplementary material for: The Maastricht Ultrasound Shoulder pain trial (MUST): Ultrasound imaging as a diagnostic triage tool to improve management of patients with non-chronic shoulder pain in primary care
Source: BMC Musculoskelet Disord. 2011 Jul 8;12:154. doi: 10.1186/1471-2474-12-154 (PMC3141612; doi:10.1186/1471-2474-12-154)
Supplement: Additional file 1 — Ultrasound examination technique and diagnosis. Specification of the US technique and the criteria for diagnosis [file 1471-2474-12-154-S1.DOC]

**Additional file 1**

**Ultrasound examination technique and diagnosis [22-26]**

| **Positioning of the patient** |
| --- |
| The patient will be seated on a rotating stool. The elbow will be positioned in 90° flexion with the hand unsupported in supination (neutral position). The patient’s arm will be placed in a series of positions to allow optimal assessment of all structures. |
| **Structures and scan position** |
| The examination includes evaluation of the biceps, subscapularis, supraspinatus, infraspinatus, and teres minor tendon; the acromioclavicular (AC) joint; the subacromial-subdeltoid (SS) bursa; and posterior labrum. Each tendon will be assessed by means of scanning planes oriented according to their long- and short-axis, and from their myotendinous junction to the bony insertion. Hyperemia/neovascularity will be semiquantitatively assessed and divided into four grades (0-3) with color doppler.  Long head of the biceps tendon Arm in neutral position.  Subscapularis tendon Arm in neutral position and during external rotation.  Supraspinatus tendon Arm behind patient’s back, with the hand close to the opposite scapular tip (Crass position). If this position is not possible patient’s hand palm will be placed on the superior aspect of the iliac wing with the elbow flexed, directed posteriorly and toward the midline (Modified Crass or Middleton position).  Infraspinatus tendon Forearm in supination on the ipsilateral thigh or across the front of patient’s chest, with the hand resting on the opposite shoulder. Visualization will be enhanced by passive internal and external rotation, during real-time dynamic imaging.  SS bursa Arm in neutral position, also with the patient’s arm behind his of her back and during testing for subacromial impingement.  AC-joint Arm in neutral position with the transducer in a coronal plane, also shifting and rotating the transducer over the acromion.  Subacromial impingement The transducer will be placed coronally with its medial margin at the lateral margin of the acromion. The patient will abduct his arm while it is in internal rotation. The supraspinatus tendon and SS bursa should glide easily under the acromion until the greater tuberosity nearly touches it. Next, this procedure will also be performed during flexion, with the transducer placed sagittally with its posterior margin at the anterior margin of the acromion. |
| **Criteria for diagnosing pathology** |
| In general, secondary ultrasound signs, such as greater tuberosity cortical irregularity, joint and/or bursal fluid, are valuable in the diagnosis of rotator cuff tears. All ultrasound examinations are interpreted prospectively and digital stored. The length and width or degree of retraction of a tear, when present, will be measured.  Tendinopathy The tendon is locally or diffusely swollen and has a heterogeneous hypoechoic appearance.  Calcific tendinitis The tendon contains hyperechoic foci. Three main types of calcium deposits can be identified. hyperechoic foci with a well-defined acoustic shadow (Type I); hyperechoic foci with a faint (Type II) or absent shadow (Type III). Dynamic examination to reveal impingement and local hyperemia will be assessed  Full-thickness rotator cuff tear Any of the following criteria is present: focal thinning of the tendon, complete non-visualization of the tendon, focal discontinuity in the homogeneous echogenicity of the tendon without focal thinning, or inversion of the superficial bursa contour and/or hyperechoic material in the location of the tendon that fails to move with the humeral head during real-time dynamic imaging.  Partial-thickness rotator cuff tear Any of the following criteria is present: a hypoechoic discontinuity in the tendon in which the lesion involves either the bursal or articular side of the tendon, or a mixed hyperechoic and hypoechoic region within the tendon. The area does not change its appearance on short- and long-axis scans and while tilting the transducer over the tendon.  Biceps tendon tear The tendon contains respectively an anechoic cleft  (partial or complete) and fluid in the sheath or a complete anechoic cleft through the tendon or a fluid-filled sheath separating the retracted ends of the torn tendon.  SS bursitis A thin hypoechoic layer of more than 2mm sandwiched between hyperechoic peribursal fat, in turn sandwiched between the hypoechoic deltoid muscle and supraspinatus tendon with hyperemia and/or a teardrop-shaped structure with the most distended segment of the bursa most distal and most dependent. Fluid in the bursa has also to be considered as a secondary sign of a partial-thickness rotator cuff tear at the bursal side or a full-thickness tear.  Subacromial impingement The supraspinatus tendon catches on or bunches up lateral or anterior to the acromion. The presences of bursal thickening without a history of inflammatory arthropathy and/or movement of fluid into the most lateral portion of the SS bursa during abduction are secondary signs of impingement.  AC osteoarthritis Any of the following criteria is present: narrowing of the joint space, irregularities and/or osteophytes of the articular bone surfaces, and/or para-articular cysts  Os acromiale Well defined cortical discontinuity of the superior aspect of the acromion, often mimicking a double AC-joint. |
