# Supplementary material for: The Maastricht Ultrasound Shoulder pain trial (MUST): Ultrasound imaging as a diagnostic triage tool to improve management of patients with non-chronic shoulder pain in primary care
Source: BMC Musculoskelet Disord. 2011 Jul 8;12:154. doi: 10.1186/1471-2474-12-154 (PMC3141612; doi:10.1186/1471-2474-12-154)
Supplement: Additional file 2 — Ultrasound diagnosis tailored treatment steps. specification of the evidence based tailored treatment steps [file 1471-2474-12-154-S2.DOC]

**Additional file 2**

**Ultrasound diagnosis tailored treatment steps**

| **Tendinopathy** | | | | | |
| --- | --- | --- | --- | --- | --- |
| **Step 1** | | **Physical therapy protocol**[21, 31] | | | |
|  |  | **Content** | An evidence based exercise protocol. Initially, supervised exercises with  manual therapy is recommended. During that time, patients should be  instructed in a home program.  -Exercises: range of motion, flexibility, strengthening , scapular stabilization  -Manual therapy: joint and soft tissue mobilization techniques augment the effect of the exercise program. | | |
|  |  | **Duration** | 6-12 weeks | | |
|  |  | **Evaluation** | In case of insufficient improvement, step 2 is advised. | | |
| **Step 2** | | **Re-consultation GP** | | | |
|  |  | **Content** | Treatment will be left to the discretion of the GP (possible options: wait-and-  see policy; subacromial corticosteroid injection; referral to an orthopedic  surgeon) | | |
| **Calcific tendinitis** | | | | | |
| **Step 1** | | **Subacromial corticosteroid injection**[4, 9, 30] | | | |
|  |  | **Content** | | | Subacromial corticosteroid injection with a mixture of 1 ml triamcinoloni acetonidum 40 mg/ml and 4-9 ml lidocaine 10 mg/ml. |
|  |  | **Procedure** | | | Posterolateral entry approach to the subacromial region. |
|  |  | **Evaluation** | | | In case of insufficient improvement after 2-4 weeks, a second injection can  be given or step 2 is advised. |
| **Step 2** | | **Re-consultation GP** | | | |
|  |  | **Content** | | | Treatment will be left to the discretion of the GP (possible options: wait-  and-see policy; referral to a physiotherapist or an orthopedic surgeon) |
| **Subacromial-subdeltoid bursitis** | | | | | |
| **Step 1** | **Subacromial corticosteroid injection**[9, 28, 36] | | | | |
|  | **Content** | | | Subacromial corticosteroid injection with a mixture of 1 ml triamcinoloni acetonidum 40 mg/ml and 4-9 ml lidocaine 10 mg/ml. | |
|  | **Procedure** | | | Posterolateral entry approach to the subacromial region. | |
|  | **Evaluation** | | | In case of insufficient improvement after 2-4 weeks, a second injection can be given or step 2 is advised. | |
| **Step 2** | **Re-consultation GP** | | | | |
|  | **Content** | | | Treatment will be left to the discretion of the GP (possible options: wait-and-see policy; referral to a physiotherapist or an orthopedic surgeon) | |

| **Partial-thickness tear** | | | |
| --- | --- | --- | --- |
| **Step 1** | **Physical therapy**[33, 34] | | |
|  | **Content** | These patients are treated similarly to those with tendinopathy. | |
|  | **Duration** | 6-12 weeks | |
|  | **Evaluation** | In case of insufficient improvement, step 2 is advised. | |
|  | **Exception** | Acute tears are immediately discussed with an orthopedic surgeon. | |
| **Step 2** | **Re-consultation GP** | | |
|  | **Content** | Treatment will be left to the discretion of the GP with the advise to  administer a subacromial corticosteroid injection or to consult  an orthopedic surgeon. | |
| **Full-thickness tear** | | | |
| **Step 1** | **Referral to an orthopedic surgeon**[9, 17, 27, 29, 32, 35] | | |
|  | **Content** | | Treatment will be left to the discretion of the orthopedic surgeon. |
| **Step 2** | **Physical therapy (in case step 1 is not executed)** | | |
|  | **Content** | | These patients are treated similarly to those with tendinopathy. |
